# A database of groundwater wells in the United States

**DOI:** 10.1038/s41597-024-03186-3

**Published:** 2024-04-04

**Authors:** Chung-Yi Lin, Alexandra Miller, Musab Waqar, Landon T. Marston

**Affiliations:** Department of Civil and Environmental Engineering, Virginia Tech, Blacksburg, VA USA

**Keywords:** Water resources, Civil engineering, Hydrology, Environmental impact, Hydrogeology

## Abstract

Groundwater wells are critical infrastructure that enable the monitoring, extraction, and use of groundwater, which has important implications for the environment, water security, and economic development. Despite the importance of wells, a unified database collecting and standardizing information on the characteristics and locations of these wells across the United States has been lacking. To bridge this gap, we have created a comprehensive database of groundwater well records collected from state and federal agencies, which we call the United States Groundwater Well Database (USGWD). Presented in both tabular form and as vector points, USGWD comprises over 14.2 million well records with attributes, such as well purpose, location, depth, and capacity, for wells constructed as far back as 1763 to 2023. Rigorous cross-verification steps have been applied to ensure the accuracy of the data. The USGWD stands as a valuable tool for improving our understanding of how groundwater is accessed and managed across various regions and sectors within the United States.

## Background & Summary

Groundwater plays a crucial role in the terrestrial hydrologic cycle, acting as a significant component in maintaining natural systems and human livelihoods. Groundwater serves not just as an important water source, contributing to drinking water, irrigation, and industrial needs, but also is integral in the provisioning of ecosystem services. However, the sustainability of groundwater is increasingly threatened by overexploitation, contamination, and the impacts of climate change^1–4^. A comprehensive understanding of the spatial and temporal dynamics of groundwater, including its extraction, within this broader hydrologic context is essential for its effective management. Central to this understanding is the need for reliable data on groundwater wells, which, beyond being mere access points to aquifers, are critical in understanding the overall health and functionality of these water systems. Despite the significance of groundwater wells, there is a lack of a cohesive, national-scale dataset in the United States that integrates and standardizes diverse state-level data. This paper aims to address this deficiency by presenting a comprehensive dataset of groundwater well records across the United States, compiled from state-level data sources and standardized using a new data model, thereby contributing to a more holistic view of groundwater management and access.

**Fig. 1:**
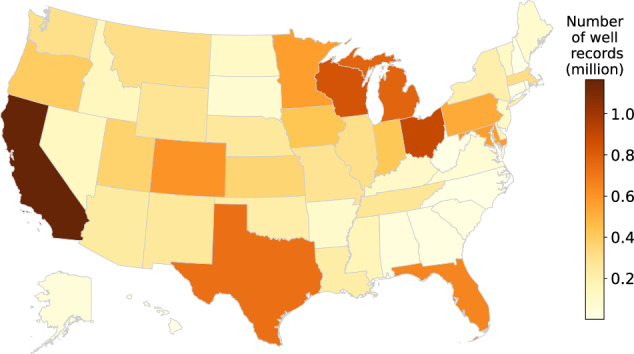
Number of groundwater well records reported by each state after cleaning and standardization (i.e., USGWD–Tabular). In total, the data product contains 14,260,752 well records (4,210 records were removed from the raw data during the cleaning and standardization process, as described in the following sections).

**Fig. 2:**
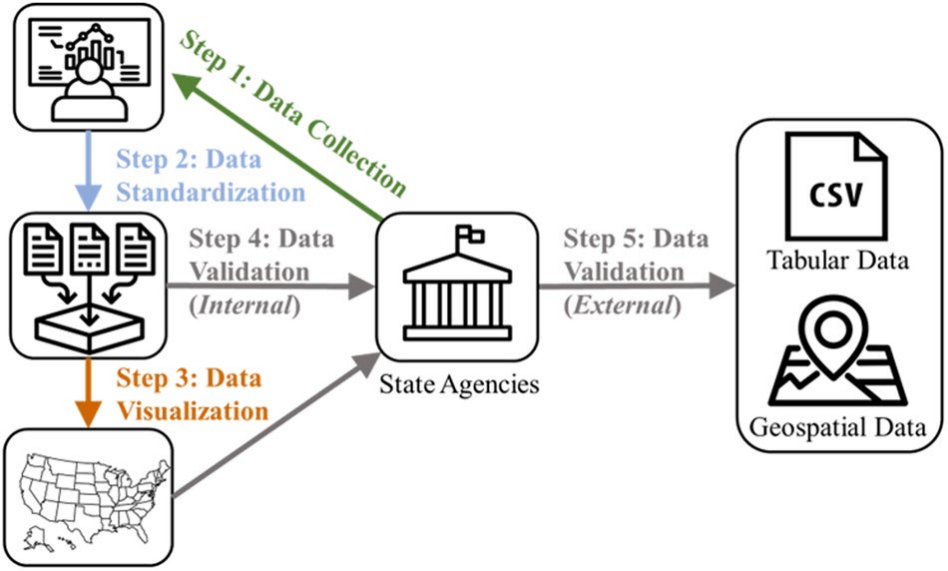
Outline of the process to develop the United States Groundwater Well Database (USGWD). Data was collected from state agencies (Step 1) and then harmonized using our data standard (Step 2). The data was then visualized (Step 3) to help identify erroneous data, as well as other features and issues with the data (Step 4). Lastly, data was reviewed by members of the research team (Step 4) and state agencies (Step 5).

In this article, we create a novel groundwater well data product named the *United States Groundwater Well Database (USGWD)*. This data product was developed using a new data standard designed to standardize heterogeneous groundwater well data collected from various sources. The harmonized dataset (i.e., USGWD) was validated by state authorities and subjected to quality assurance and quality control (QA/QC) procedures to verify the spatial accuracy of the well locations. We also performed a comparison between our assignment of irrigation subcategories using well locational information and state-assigned irrigation subcategories. This paper presents two groundwater well datasets: i) *USGWD–Tabular:* a tabular inventory detailing locational information, water uses, operational status, and other attributes of each well; ii) *USGWD–Geospatial:* geospatial data depicting the location of collected groundwater well records that pass our locational QA/QC. Figure 1 depicts the number of groundwater well records reported by each state after data cleaning and standardization (i.e., USGWD–Tabular). An overview of the development of USGWD, including detailed descriptions of the datasets, are in Fig. 2 and Table 1.

**Table 1:** Overview of the two groundwater well data products. Both data products can be found at the HydroShare data repository^11^.

Data Products	Description	Data Format
United States Groundwater Well Database–Tabular (USGWD–Tabular)	Tabular data detailing groundwater well records, including their locational information, water uses, operational status, and other attributes of each well.	CSV
United States Groundwater Well Database–Geospatial (USGWD–Geospatial)	Geospatial data showing the location of each groundwater well record, where entries with missing coordinates or coordinates that do not match the state-provided county or state are removed.	Esri Shapefile (SHP)

## Methods

We define a groundwater well as a man-made excavation equipped for either extracting or monitoring water from an aquifer. Our database, USGWD, encompasses all records of groundwater wells in the United States that can be made publicly available. Well records are independently collected and maintained by each state, leading to variations in coverage, duration, completeness, and quality of the records from state to state. These variances are mirrored in USGWD, as it compiles data from these diverse state sources. To address differences in state reporting, our database converts disparate state records into a standardized format, featuring a common set of attributes that are consistently reported across most states. This includes details on well infrastructure, location, operational status, water source, and primary water use. However, data on water quality, lithology, and withdrawal rates, which are not consistently reported by all states, are not included in USGWD due to their limited availability.

The development of USGWD included three steps: (i) data collection, (ii) data standardization, and (iii) data validation, which are detailed in the sections that follow.

### Data collection

In developing a comprehensive national groundwater well dataset, our first step involved identifying potential data sources based on the work of Perrone and Jasechko^5^ and Murray *et al*.^6^ who provided a list of various state organizations that collected and maintained groundwater well information. Unfortunately, direct access to the data compiled by these researchers was not available to us during our research. Second, we undertook an examination of the publicly accessible data repositories and state websites flagged by these previous studies. This review guided our efforts to acquire groundwater well data from the remaining states directly from each state by contacting the designated state representatives, which would occasionally lead to additional data sources not listed by either Perrone and Jasechko or Murray *et al*. Our outreach strategy primarily employed emails and phone calls as means of communication. In instances where these approaches did not yield the required data, we resorted to submitting requests under the Open Records Act or the Freedom of Information Act (FOIA) to secure the necessary records. This process resulted in 23 states supplying data from private datasets (Table 2) and 32 states providing well data through publicly accessible websites (Table 3). Note that some states provided data online, while also maintaining additional well records that had not been published, and therefore show up in both Tables 2, 3. A summary of our data sources is also provided in Table S1.

**Table 2:** State agencies that provided private datasets for the United States Groundwater Well Database.

State	Organization managing well data	Ref
Alaska	Alaska Department of Natural Resources	^16^
Arkansas	United States Geological Survey-Lower Mississippi Gulf Water Science Center	^17^
California (Part 1)	California Department of Water Resources	^18^
Connecticut	Connecticut Department of Energy & Environmental Protection	^19^
Florida (Northwest)	Northwest Florida Water Management District	^20^
Florida (South)	South Florida Water Management District	^21^
Florida (St. John’s River)	St. Johns River Water Management District Bureau of Water Supply Planning	^22^
Florida (Suwannee River)	Suwannee River Management District	^23^
Georgia	Georgia Department of Natural Resources	^24^
Hawaii	State of Hawaii, Department of Land and Natural Resources	^25^
Illinois	Illinois State Geological Survey	^26^
Iowa (Part 1)	Iowa Department of Natural Resources	^27^
Kansas (Part 1)	Kansas Geological Survey	^28^
Maryland	Maryland Department of the Environment	^29^
Mississippi	Mississippi Department of Environmental Quality	^30^
New Hampshire	New Hampshire Department of Environmental Services	^31^
New Jersey	New Jersey Department of Environmental Protection	^32^
New York	New York Department of Environmental Conservation	^33^
North Carolina (Part 1 & 2)	North Carolina Department of Environmental Quality	^34,35^
Ohio	Ohio Department of Natural Resources	^36^
Rhode Island	Rhode Island Department of Health	^37^
South Carolina (Part 2)	South Carolina Department of Natural Resources	^38^
Vermont	Vermont Department of Environmental Conservation	^39^
Virginia	Virginia Department of Environmental Quality	^40^
West Virginia	West Virginia Department of Environmental Protection	^41^
Wisconsin	Wisconsin Department of Natural Resources	^42^

**Table 3:** Names and source links for states offered public datasets contributing data to the United States Groundwater Well Database.

State	Organization managing well data	Ref
Alabama	Geological Survey of Alabama	^43^
Arizona (Part 1 & 2)	Arizona Department of Water Resources	^44,45^
California (Part 2)	California Department of Water Resources	^46^
Colorado	Colorado Division of Water Resources	^47^
Delaware	Delaware Department of Natural Resources and Environmental Control	^48^
Florida (Southwest)	Southwest Florida Water Management District Water Supply Section	^49^
Idaho	Idaho Department of Water Resources	^50^
Indiana	Indiana Department of Natural Resources	^51^
Iowa (Part 2)	Iowa Department of Natural Resources Water Supply Section	^52^
Kansas (Part 2)	Kansas Geological Survey	^53^
Kentucky	Kentucky Geological Survey	^54^
Louisiana	Louisiana Department of Natural Resource	^55^
Maine	Maine Geological Survey	^56^
Massachusetts	Water Management Program, MassDEP	^57^
Michigan	Michigan Department of Environment, Great Lakes, and Energy	^58^
Minnesota	Minnesota Department of Health - Environmental Health Division - Source Water Protection Unit	^59^
Montana	Montana Bureau of Mines and Geology	^60^
Missouri	Missouri Spatial Data Information Service	^61^
Nebraska	Nebraska Department of Natural Resources	^62^
Nevada	State of Nevada Division of Water Resources	^63^
New Mexico	New Mexico Office of the State Engineer	^64^
North Dakota	North Dakota Department of Water Resources	^65^
Oklahoma	Oklahoma Water Resources Board	^10^
Oregon (Part 1 & 2)	Oregon Water Resources Department	^66,67^
Pennsylvania	Pennsylvania Department of Conservation & Natural Resources	^68^
South Carolina (Part 1)	South Carolina Department of Natural Resources	^69^
South Dakota	South Dakota Department of Agriculture & Natural Resources	^70^
Tennessee	Tennessee Department of Environment & Conservation	^71^
Texas	Texas Water Development Board	^72^
Utah	Utah Geospatial Resource Center	^73^
Washington	Washington State Department of Ecology	^74^
Wyoming	Wyoming State Engineer’s Office	^75^

We directly communicated with state officials who offered insights into the methods of data collection, definitions, and the interpretations of any notations utilized. State officials also provided us with associated metadata, any disclaimers, details about publishing restrictions, and assessments regarding how representative their data was for their state. Out of all the states, only 20 could furnish an estimate of the completeness of their well records. Most states acknowledged that their records might be incomplete, particularly for older wells that were established before their records began. Generally, most states mandate the reporting of water use wells, with variations in requirements based on the well’s drilling date (with older wells being less frequently reported), capacity (with larger wells more commonly reported), and purpose (with domestic wells being reported in nearly all states). Therefore, USGWD is more complete for some well classes than others, though the exact level of completeness is difficult to estimate (see estimates by state officials in Table S2).

Many states noted that they continuously receive information about new and existing wells, meaning that our study only captures the most up-to-date data available at the time of our data collection. Moreover, several states indicated that their records are not routinely updated, and any unreported modifications to wells would not be reflected in their data (e.g., some inactive wells may still be reported as active in state records). Table S2 in our study details the date when data was collected from each state, alongside the contact information of the primary official and organization, disclaimers, and the estimated completeness of their records. Our data collection spanned from August 2021 to January 2024. It’s important to note that any well records added to a state’s repository after our collection dates are not included in USGWD.

All states granted us explicit permission to publish the groundwater well records they provided to us and were given multiple opportunities to review the representation of their data in USGWD. However, five states stipulated that we suppress information such as owner names, addresses, and phone numbers. In compliance with these requests, we excluded owner information, such as names and contact details, from USGWD for all states. Nonetheless, we were permitted to incorporate the geographical coordinates of each groundwater well records in the database for every state.

### Data standardization

The data formats and types received from various states differed significantly. In USGWD, we ensured uniform definitions and representations for all groundwater well records. Though the initial focus of our study was to identify pumping wells, most states also provided us with records on monitoring and other well types. We have included these records within USGWD and labeled them in accordance with their purpose.

We established attribute names and definitions to categorize commonly reported data into standardized groups. The attributes incorporated in USGWD include Well ID, Well ID (State), Longitude, Latitude, County, State, FIPS, Aquifer-Specific, Aquifer-Broad, Subwatershed-HUC12, Subwatershed-Name, Location Verified, Flag County, Flag State, Flag US, Well Depth (Feet), Screen Depth (Feet), Length of Screen (Feet), Well Capacity (GPM), Lithological Data, Surface Elevation (Feet), Status, Year Well was Constructed, Year Reported, USGS Water Use Category, Irrigation Subcategory (State), Irrigation Subcategory (Land Use), Water Quality (Potable/Non-Potable), and Flag Duplicate. The exact definitions of these attributes are detailed in Table S3. To account for the variations in how different states define well attributes, our dataset uses broad definitions for each attribute. We also provide a crosswalk table in Table S4, which aligns the original attribute names from each state with those used in our database, facilitating a comprehensive understanding of the data across different state systems.

We mapped attributes in the state records to the attribute definitions specified by our data standard. State agencies provided official documents outlining the definitions of attributes in their records, along with explanations for any abbreviations and labels used. In cases where such documentation was not available, the officials lent their expertise to define the features within the dataset. Data attributes most commonly reported across all states were incorporated into USGWD. Consequently, some well attributes that were collected are not included in USGWD.

We use broad water use categories (Table S5) to accommodate for variations in state definitions. The naming conventions for these categories are primarily taken from the United States Geological Survey (USGS) water use definitions^7^, with some necessary modifications and additions. Specifically, the categories of Irrigation-Crop (IR-C), Irrigation-Golf Courses (IR-G), Irrigation-Unknown (IR-U), Remediation (RM), and Other slightly diverge from the original USGS definitions. Additionally, we introduced a category named “Monitor (MO)” to signify wells used for monitoring purposes. The three irrigation subcategories (IR-C, IR-G, and IR-U) provide more specific distinctions within the more general Irrigation (IR) category.

We classified the uses of each groundwater well into our standardized water use categories with the assistance of state officials and utilizing the documentation gathered during data collection (Table S6). In cases where a well serves multiple functions, each purpose is separately recorded in USGWD. Achieving a perfect one-to-one match between the different state water use definitions and our study’s definitions was not always feasible, occasionally leading to a many-to-one or one-to-many mapping in our database. This may give the perception that a well has multiple uses, even if it is dedicated to a single purpose. For instance, a well labeled as ‘Agriculture’ in a state might encompass both irrigation and livestock applications. In the USGWD, such a well would be classified under both Irrigation (IR) and Livestock (LV) uses. However, it is possible that the well is only used for crop irrigation, not for livestock, or vice versa.

In our data standard, the general label Irrigation (IR) is used to indicate wells primarily for irrigation when specific details about what is being irrigated are not provided. The subcategories within Irrigation, Irrigation-Crop (IR-C), Irrigation-Golf Courses (IR-G), and Irrigation-Unknown (IR-U), are employed for well records with clearly defined irrigation purposes. A well record categorized into one of these subcategories is also included in the broader Irrigation (IR) category. Furthermore, we assign the classification ‘Unknown’ to records where the water uses are unspecified or unclear, covering a range of statuses like unknown, unused, destroyed, decommissioned, abandoned, and reused. The IR-U category also often includes irrigation of parks, lawns, cemeteries, and other irrigation purposes not captured by IR-C or IR-G.

We employ an automated screening to identify duplicate records that share identical Well ID (State), Longitude, and Latitude attributes. These potentially duplicate records were flagged (Flag Duplicate) but not removed from USGWD. While these entries are likely duplicates, there could be instances where a common owner operates multiple wells for the same purpose on a property and the same Well ID and coordinates were given for all wells (e.g., coordinates of the centroid of property or property entrance). Some states report the geographic coordinates of the well as being at the center of the quarter section (e.g., Oregon) or section (e.g., California) that the well is located within, potentially leading to multiple wells with the same coordinates. With a few exceptions, as noted below, states were often unsure whether these well records were duplicates or unique. To safeguard the integrity of the database and to limit subjective or arbitrary changes to the data, we opt to flag potential duplicate records (i.e., those with identical Well ID (State), Longitude, and Latitude) rather than eliminate them when it was unclear whether a record was a duplicate. There are 1,421,235 well records out of 14,260,752 in USGWD that were flagged as potential duplicate records.

Duplicate records were removed only when states advised us on how to identify and remove duplicate records or when sufficient evidence existed to give us confidence the records were in fact duplicates. Two states informed us of duplicate records and helped us to process those records accordingly. In New Jersey, wells were recorded across different phases, including permit, record, and decommissioning phases. For decommissioned wells, only the decommissioning entry was included in our study, while for wells that had both permit and record entries, the permit entry was excluded. In Northwest Florida, wells were recorded when they were constructed and when they were decommissioned. In cases where both entries existed for a single well, the constructed entry was excluded, and the decommissioned entry was included in our study. A similar logic was used for other states as well. That is, when records are nearly identical but have a different activation status or owner name (indicating a change in operation or owner), the most recent record is maintained within USGWD. If records were not dated, both records were maintained in USGWD but flagged as possible duplicates.

In our dataset, some states used unique abbreviations or codes to denote the county of a well’s location. We converted these various abbreviations and codes into their corresponding full county names to maintain consistency across the entries from different states. A list of the original county codes and abbreviations provided by the states, which were then converted, can be found in Table S7. Additionally, each well record was assigned a Federal Information Processing System (FIPS) code, corresponding to the state and county where the well is located, based on the reported coordinates. However, there are occasions where the state county assignment may differ from the county assignment based on the well’s coordinates. To address this, any records exhibiting inconsistencies in the reporting of well locations are marked within USGWD with a specific flag (i.e., Flag County). This flagging helps to identify and acknowledge instances of potential discrepancies in the dataset.

States have different ways of reporting if a well (record) is active (currently being used or capable of doing listed well purposes) or inactive (not currently being used). For some states that did not directly report the well’s status within their well database, they offered us guidance on classifying wells as active or inactive using other data available within the dataset. For records where the status of the well remained ambiguous, we assigned the designation “Unknown” within our dataset. To facilitate understanding and comparison, we have included a crosswalk table (Table S8) that links well activation statuses assigned by the states to the classifications used in our dataset.

States provided different well screen information. Typically, states documented the start and end points of the well screen, measured in feet from the land surface, or they provided the total length of the screen. In cases where the length of the well screen was not explicitly provided, we calculated it by deducting the depth at which the screen began from the depth at which it ended.

Well coordinates were in varying formats and datum across states and even within states. Our procedure involved converting all coordinates to the NAD83 datum in decimal degrees, with manual corrections for clear errors. For instance, we rectified missing negative signs in longitudes or misplaced decimal points (e.g., converting latitude 4118.8924 to 41.188924). If datum information is not available, we assumed the coordinates are from NAD83 datum. Lastly, coordinates located outside of the world range (i.e., latitudes not in [−90, 90] or longitude not in [−180, 180]) are replaced with nan.

We identified the potential aquifer and the Hydrologic Unit Code (HUC12) boundary that overlapped with each well record. We overlaid aquifer^8^ and HUC12^9^ shapefiles with the groundwater well locations. The aquifer geodatabase we utilized includes 440 major and minor aquifers across the U.S^10^. However, it’s worth noting that some smaller or less researched aquifers might not be represented in this database, raising the possibility that a well could be drawing water from an aquifer not listed in USGWD. Twenty-three states provided at least some information about the aquifer accessed by different wells, but disparate reporting approaches and naming conventions made it difficult to compare these to other sources. As a result, our database was enhanced to include the name of the broad and specific aquifer underlying the well, as well as the 12-digit HUC code and the corresponding name of the area where the well is situated.

## Data Records

The USGWD consists of a csv file (USGWD–Tabular) and a shapefile (USGWD–Geospatial) for each state. Additionally, metadata is provided in Table S1–S10, including:

- Table S1-Name of the state organization that provided the data and the corresponding state agency’s website.
- Table S2-Summary table of when the data was collected, the person and organization that primarily assisted with the data collection, disclaimers, and representation estimations for each state.
- Table S3-Definitions of attributes in our dataset.
- Table S4-Crosswalk between the state dataset attributes to the corresponding attributes in USGWD.
- Table S5-Water use categories assigned to each well and their corresponding definitions.
- Table S6-Crosswalk between state assigned water use category for groundwater wells to the corresponding USGWD water use category.
- Table S7-Crosswalk between state assigned county labels to the full name of each county.
- Table S8-Crosswalk between state assignments of active and inactive wells to our corresponding assignments.
- Table S9-Crosswalk between state assignment of how a well location is obtained to our corresponding categorization of whether the well has been GPS or field located.
- Table S10-Crosswalk of eligible crops classified as croplands in USGWD.

The Python code used for data standardization is also provided. All data, metadata, and code can be found at the HydroShare data repository^11^ (10.4211/hs.8b02895f02c14dd1a749bcc5584a5c55) under CC BY license.

New groundwater wells are continually drilled, and old wells abandoned. However, USGWD currently represents a static data product that captures all known wells around the time of its publication. A significant obstacle in maintaining an up-to-date database is the variability in public data access across states. While some states readily release their data, others necessitate FOIA requests, a process often mired in bureaucratic delays. Furthermore, the inconsistency in how regularly different states update their databases poses a challenge in keeping USGWD up to date. Despite these hurdles, the potential benefits of a dynamic, regularly updated database are substantial. It would significantly contribute to ongoing research and societal needs but would likely require the resources of a federal agency, such as USGS, to continually maintain such a data product.

## Technical Validation

The 14.2 million plus well records in USGWD significantly surpasses the number of well records reported by previously developed well databases, such as the USGS National Groundwater Monitoring Network’s (USGS, n.d.) approximately 18 thousand water-level monitoring wells, the approximately 7.6 million well records identified by Degnan *et al*.^12^, the US EPA’s documentation of 1.4 million domestic well records across 20 states^6^, and the 11.8 million well records collected by Perrone and Jasechko^5^. Besides USGWD providing a more complete accounting of US groundwater well records than previous efforts, our study is the first to publicly release a nationwide, standardized data product describing both use and monitoring wells. A direct comparison of datasets remains challenging, as none of these previous studies have made their data publicly available for a detailed comparison. The one exception is the National Groundwater Monitoring Network. Though USGWD has over 1 million more monitoring wells than the National Groundwater Monitoring Network, there are a few thousand monitoring wells in the National Groundwater Monitoring Network not included in USGWD. The USGWD does not replace, but compliments, the National Groundwater Monitoring Network, as the latter has detailed time-series data that is not reported by USGWD.

Our dataset went through multiple rounds of quality assurance and quality control (QA/QC) to ensure its integrity before finalization. A key focus of the QA/QC process was on verifying the accuracy of the location of the groundwater wells. Typically, well records were provided to the states by either the owner or the well driller. Records that were either obtained using a global positioning system (GPS) device or field-verified by staff from the collecting agency were deemed to have greater locational accuracy compared to those without such verification. To reflect this in our dataset, we included a column that indicates the level of location verification for each well record. This categorization helps users understand the reliability of the locational data. Records without coordinates were labeled as ‘Unknown’ in the Location Verified column. The specific state labels used for these verification classifications are detailed in Table S9. It is important to note that well locations not recorded by GPS are not necessarily incorrect. Those records may simply have not undergone state verification but still be accurate. In other cases, states may use several other methods to verify a well’s location. However, these approaches may only confirm that the well is on the correct property (not its precise location), or the method may involve greater subjectivity (e.g., matching the name on a mailbox or confirming based on information from a neighbor).

Data entry inaccuracies can occur, either by the state or by the initial reporter of the groundwater well location. To identify potential errors in the locational data, we examined the consistency between the assigned county and coordinates for each well in the state records. This involved assigning each well to a county based on whether its coordinates fell within the boundaries of that county, using a county shapefile as a reference^13^. We then compared the counties reported by the states with those determined from the coordinates of the well records. This comparison aimed to verify the accuracy of state data regarding well locations. In USGWD, records are flagged if the state-reported county and coordinate-specified county differ or are incomparable due to missing information. We also flag records that have no coordinates, or the coordinates are outside of the US border.

**Fig. 3:**
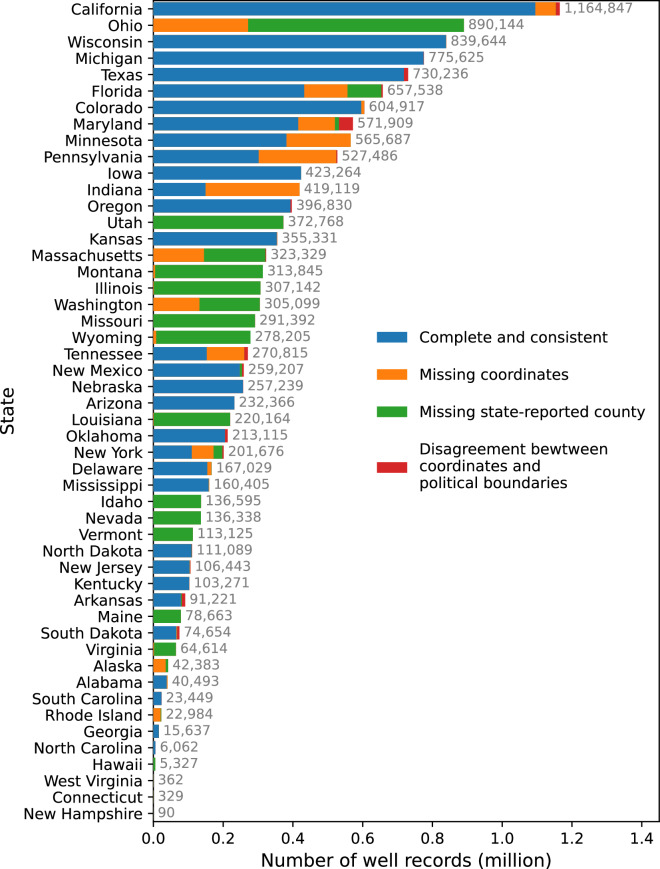
Distribution of well records by locational accuracy across states. This figure illustrates the total count of well records per state, featuring locational specifics such as coordinates and/or county information. Well records lacking reported coordinates are depicted in orange, whereas those absent of state-reported county details are in green. Records with coordinates that mismatch the state-reported county or the actual state of the well’s location are highlighted in red. All remaining well records are represented in blue.

Our collection includes over 14.2 million well records from the United States, but approximately 1.9 million of these records either lacked coordinates or were situated outside the reported state or county. Figure 3 illustrates the number of well records per state and specifies the quantity of records with missing or inconsistent locational details.

**Fig. 4:**
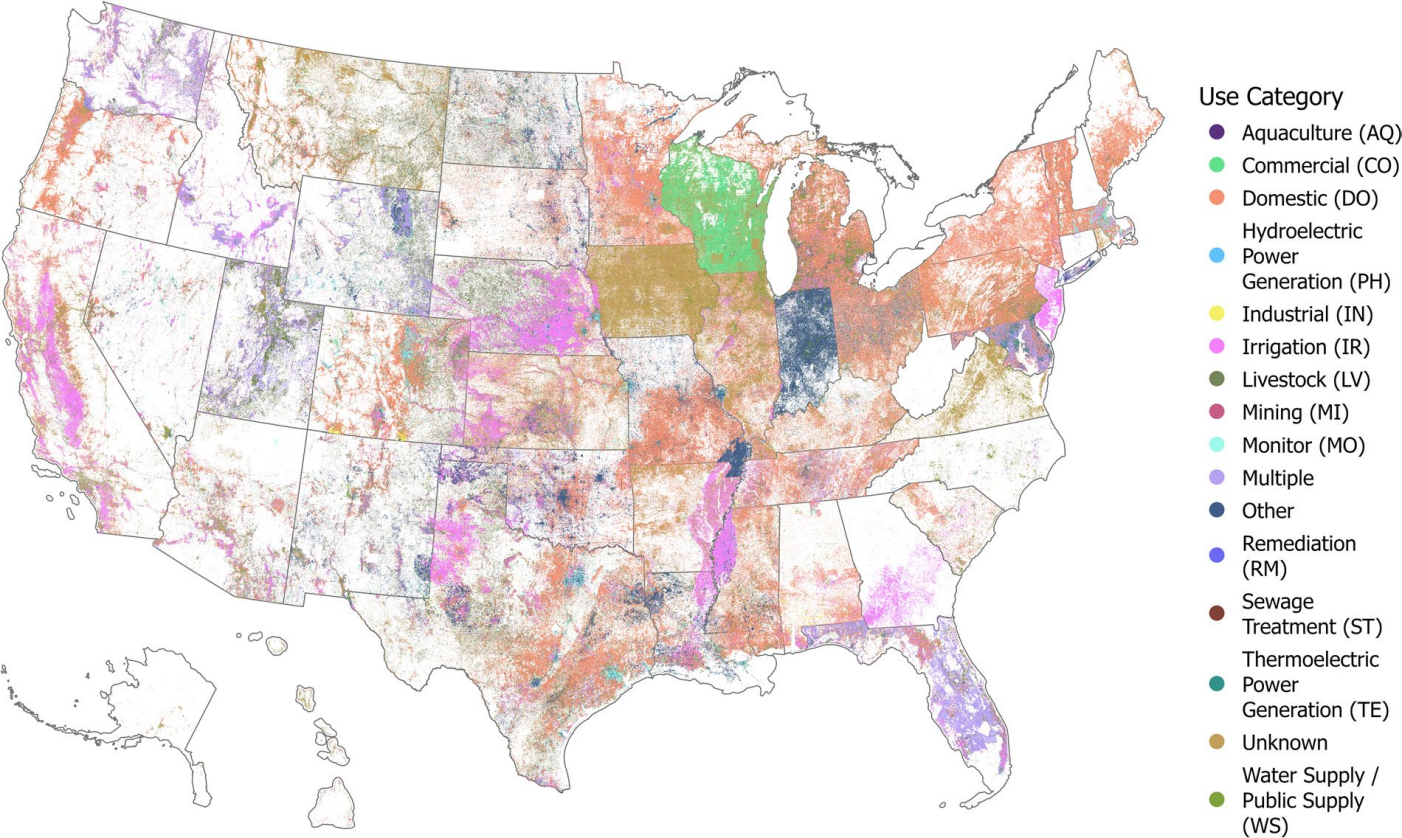
Water use types of 12,344,774 groundwater well records by use type in the United States (i.e., USGWD–Geospatial) after removing 1,915,978 records that had missing or incorrect locational details in USGWD–Tabular.

The database contains just over 12.3 million records that have consistent and complete locational data. Despite some records having missing, inconsistent, or incorrect location data, we chose to keep these in USGWD–Tabular, albeit with appropriate flags to indicate these issues. The distribution of well records with consistent and complete locational data is depicted in Fig. 4. We found that 25,807 well records had coordinates placing them outside their reported state. A significant portion of these records (67.2%) originated from Maryland, Florida, Massachusetts, and Tennessee. Maryland had the highest percentage of well records reported outside its state (1.6%). Additionally, we identified 1,246,59 well records with coordinates placing them outside their reported county. Around 67.2% of these well records were from Maryland, Texas, California, Tennessee, and Arkansas. Arkansas had the highest percentage (10.81%) of its well records located outside the reported county.

We could not perform locational checks for all well records because some of them did not have coordinates or county data. About 53.1% of the well records missing coordinates were from Ohio, Indiana, Pennsylvania, and Minnesota. Ohio had the most well records without coordinates (271,354). Rhode Island, Alaska, and Indiana had the highest percentage of well records without coordinates (91.1%, 83.4%, and 64.3%, respectively). Records from eighteen states (Alaska, Connecticut, Hawaii, Idaho, Illinois, Louisiana, Maine, Massachusetts, Missouri, Montana, Nevada, New Hampshire, Rhode Island, Utah, Vermont, Virginia, Washington, and Wyoming), did not include county names, making comparison between reported county and coordinates infeasible.

The reasons behind these data inconsistencies and incompleteness differ between states. Some states may have more restrictive data sharing laws and practices than others, inhibiting them from publishing well coordinates. The resources dedicated to collect, verify, and maintain groundwater well data vary significantly by state, and likely also plays a role in observed differences in data quality and completeness between state records. Further, differences in data reporting requirements and collection methods led to differences in state records.

After excluding well records with locational discrepancies, California emerged with the highest number of reported well records at 1,094,995, followed by Wisconsin with 838,822, Michigan with 774,524, and Texas with 718,111. On the lower end, New Hampshire reported the fewest well records, numbering only 81, with West Virginia reporting 160, Connecticut 329, and Rhode Island 1,686. As noted above, data sharing restrictions varied among the states. Consequently, the actual number of well records in many states might exceed what is captured in our database. Furthermore, states have different reporting requirements and the resources allocated for database maintenance that can contribute to significant discrepancies in the number of documented well records, which might not reflect the true number of wells in each state. Insights and feedback from each state regarding how representative each state’s well records are of all wells within the state are detailed in Table S2.

**Fig. 5:**
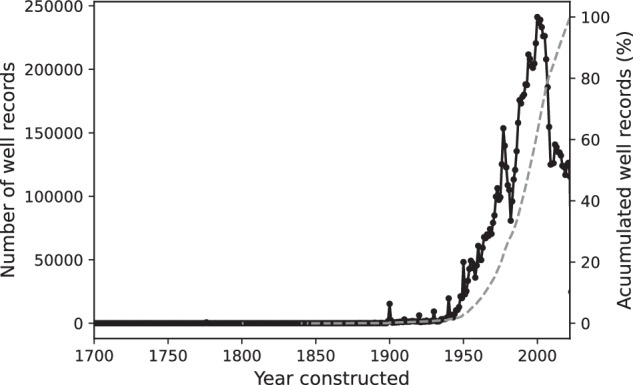
Number of wells constructed from 1700 to 2023. The accumulated number of well records in percentage is shown in a grey dashed line.

Figure 5 shows the timeline of well construction. However, nineteen states did not provide information on the construction dates of wells, and, as such, they were not included in this figure. The data shows that approximately 80% of wells were constructed between 1975 and 2023, with the year 2000 recording the highest number of wells constructed. It’s noteworthy that most states reported their current well databases only began in the 1970s. Therefore, the data for periods prior to the 1970s might not accurately represent the actual number of wells constructed due to the absence of mandatory reporting during that time. This gap in historical data necessitates caution when interpreting the construction figures for those earlier years. Post-1970s, the data reveals considerable variability in the number of wells constructed annually, with noticeable fluctuations in different years. However, the reasons for these variations in construction rates are not immediately apparent from the data alone.

**Fig. 6:**
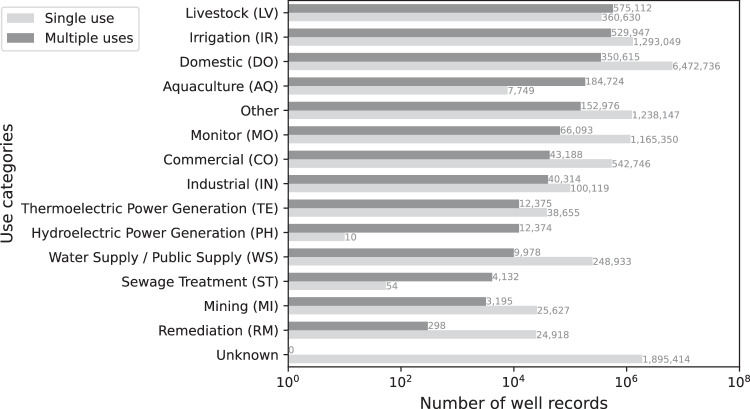
Log-distribution of well records categorized by water use type. The sum of all reported values does not equate to the total number of well records in USGWD since one well may be reported in more than one use category if it has multiple uses (i.e., dark gray bars).

Each well in USGWD was categorized into one or more water use categories based on the well use initially assigned by the reporting state. Figure 6 presents a national breakdown of well records by water use category assigned in USGWD. It’s important to note that some well records are attributed to multiple water uses and thus appear in several categories. Of the 14.2 million plus well records we analyzed, 1,895,414 records (13.3%), were categorized as ‘Unknown’ in terms of their purpose. Additionally, 846,615 records (5.9%) were assigned to multiple water use categories and 11,518,723 records (80.8%) were exclusively assigned to a single water use category.

In our study, three primary water use categories – Domestic (DO), Irrigation (IR), and Monitor (MO) – collectively represent 59.2% of the recorded uses for well water. Around 9.8% of well records were assigned a water use purpose by the state but this use classification did not fit within the water use categories we adopted for this study and the water use of these records were labeled as Other. Additionally, 13.3% of well records were categorized as ‘Unknown’ in terms of their water use. The Domestic (DO) category encompasses the largest number of well records, and wells in this category are predominantly used for domestic purposes only, signifying a single-use tendency. However, it’s interesting to note that while domestic wells outnumber irrigation wells, data from the USGS^14^ suggests that the total volume of groundwater extracted by irrigation wells is significantly higher than that from domestic wells. This highlights a disparity between the number of wells and the volume of water usage across different categories.

Since irrigation is responsible for the largest share of groundwater pumping, USGWD further distinguishes between different irrigation purposes: Irrigation-Crop (IR-C), Irrigation-Golf Courses (IR-G), and Irrigation-Unknown (IR-U). Out of the total irrigation well records, 752,219 (57.9% of all IR well records) are classified as single-use Irrigation (IR), indicating that these wells are exclusively used for a specific irrigation purpose. The remaining 42.1% of IR well records were assigned multiple irrigation purposes. While some of these records might indeed serve various irrigation functions, such as both crop irrigation and servicing golf courses, it is more common for the state’s definition of water use to be broadly defined, encompassing two or more irrigation subcategories. Therefore, a well could be classified under multiple irrigation subcategories per our definitions, but in reality, it is likely dedicated to only one specific irrigation purpose.

**Fig. 7:**
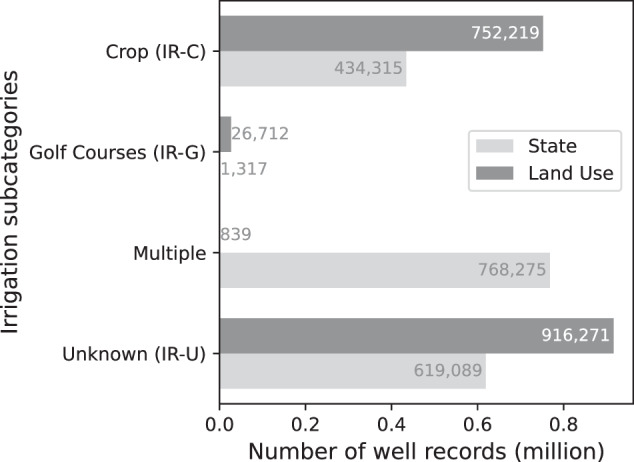
Number of well records with an irrigation purpose, broken into irrigation subcategories: Irrigation-Crop (IR-C), Irrigation-Golf Courses (IR-G), Irrigation-Unknown (IR-U), and their combinations. The State classification means the irrigation subclassification was based on the original state label. The Land Use classification used the land use corresponding to the well location to assign an irrigation subclass.

Within the single-use IR well records, 41.2% are dedicated to Irrigation-Crop (IR-C) and only 0.12% to Irrigation-Golf Courses (IR-G). The irrigation subcategory for approximately 58.7% of all IR well records are unknown (i.e., unclassified) since the state provided no details as to whether irrigation was for crop, golf, or some other purpose (Figure 7).

To further enhance the classification of irrigation subcategories, we spatially joined the well records with nationwide land parcel data from Regrid’s premium schema (https://regrid.com/). This enabled us to identify wells belonging to land parcels with irrigated cropland or golf courses. Table S10 lists the land uses labeled as croplands. If the well did not fall within a private land parcel (e.g., the well coordinates were within the easement of a nearby road), then the well was assigned to the nearest land parcel with cropland if it was less than 100 meters away from the well. The well record would then be classified as Irrigation-Crop (IR-C). Similarly, the Irrigation-Golf Courses (IR-G) category is assigned to wells falling outside a private land parcel if the nearest golf course is less than 100 meters away. Golf course boundaries were extracted from Open Street Map. If a well record meets both criteria, i.e., a well is within 100 meters of both a Regrid cropland parcel and is also within 100 meters of Open Street Map golf course boundaries, then both IR-C and IR-G are assigned since it is unclear which purpose(s) the well serves. Fig. 7 presents a comparison between the irrigation subcategories derived from state-reported water use and those we assigned using the land use classification, labelled as Land Use. To examine the alignment of the land use-based assignment to the state-based assignment, we compared the state-based irrigation well records that have coordinates to the land use-based assignments. On average, the land use-based assignments achieved 82.1% and 92.8% alignment with state-assigned IR-C and IR-G, respectively.

**Fig. 8:**
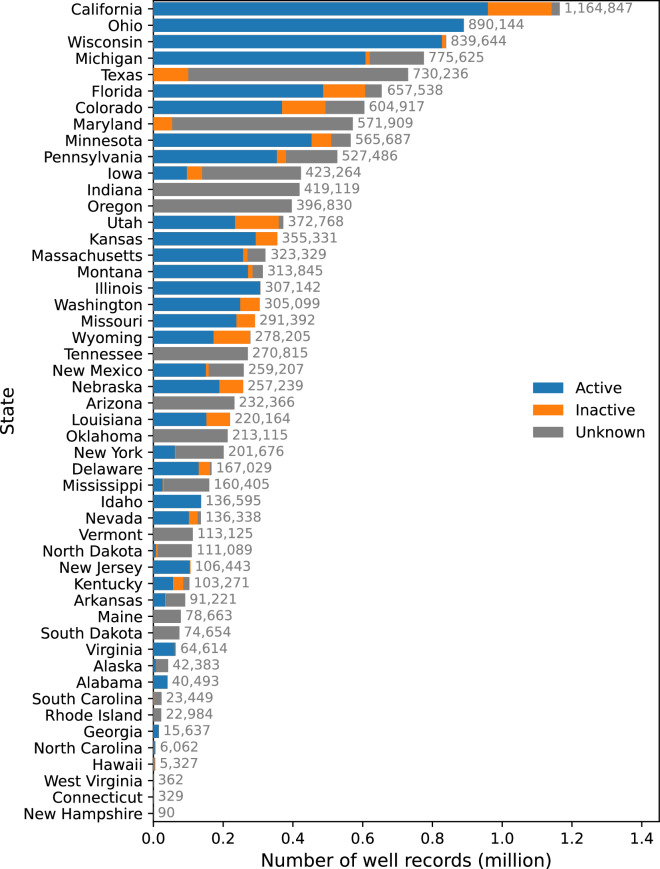
Distribution of well record status of USGWD–Tabular by state. Well records are classified as active, inactive, or unknown.

Each well record in USGWD was assigned a status of either Active, Inactive, or Unknown. Of the 14.2 million plus well records in USGWD–Tabular, 8,338,041 (58.5%) are reported as being actively used, 1,411,512 (9.9%) are inactive, and 4,511,199 (31.6%) were classified as unknown (Fig. 8). Seventy percent of reported active well records are in just eleven states: California, Ohio, Wisconsin, Michigan, Florida, Minnesota, Colorado, Pennsylvania, Illinois, Kansas, and Montana. California had the highest number of active well records with 82.3% of the well records in the state categorized as active. California also had the highest number of inactive well records; around 15.7% of its total reported well records are inactive (the status of the remaining 2% of well records is unknown). Approximately 46.6% of inactive well records were in California, Utah, Colorado, Florida, and Wyoming. Approximately 61.0% of well records with an unknown activation status were in Texas, Maryland, Indiana, Oregon, Iowa, Tennessee, and Arizona. Texas has the highest number of well records with an unknown activity status: 86.3% of the well records in Texas are categorized as having an unknown status.

Well locations were overlaid with major and minor aquifers in the United States, as identified by the United States Aquifer Database^15^, to connect well records to their underlying aquifers. Although the aquifer database we utilized is among the most comprehensive available, encompassing 440 aquifers, it does not include every aquifer in the US, particularly some smaller ones. Further, some aquifer boundaries, as well as overlapping aquifers, may not be perfectly represented. As a result, while it’s highly likely that a well is drawing groundwater from the aquifer indicated in USGWD, we cannot guarantee that the assigned aquifer is the one that the well is pumping from or monitoring. This inherent limitation in the dataset should be considered when interpreting the aquifer information associated with each well record.

Considering the potential to further enhance the utility and accuracy of aquifer mapping, an intriguing avenue for future research involves the incorporation of aquifer depth information with well screen depth provided within USGWD. While our current analysis establishes a nationally consistent characterization of groundwater wells and the major aquifer system they overlay, pairing USGWD with more detailed data on the nation’s aquifer systems, including distinctions between shallow surficial aquifers and underlying major aquifers, could serve as an enhancement to USGWD. Further, a detailed mapping of wells to aquifers would aid in modeling efforts, improving both research and water resource management and planning. However, such an endeavor faces notable challenges, including the limited availability of detailed aquifer depth data on a national scale and the complexity of many aquifer systems.

**Fig. 9:**
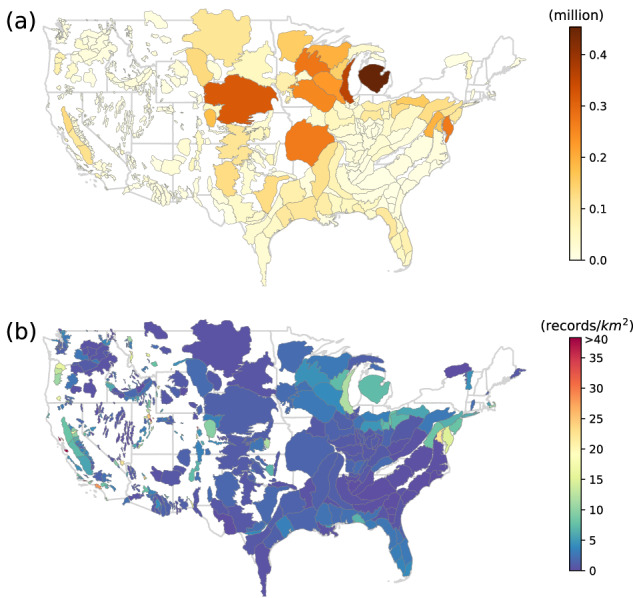
Heat maps of the a) count of groundwater well records and b) the density of well records over major and minor aquifers in the United States from USGWD–Geospatial.

Figure 9 illustrates both the quantity and density of well records overlaying each aquifer. Among the 12,344,774 well records with locational data, 9,745,274 are situated within the boundaries of aquifers as defined in the United States Aquifer Database^15^. The aquifers with the largest number of well records reported within their boundaries are the Michigan Basin (453,257), Eastern Silurian-Devonian Aquifers (357,875), and Northern High Plains (317,527) aquifers. The Santa Clara Valley and Santa Barbara and Foothill Basin aquifers had the highest density of reported well records per square kilometer at 81.7 well records per square kilometer and 54.0 well records per square kilometer, respectively. Despite the Michigan Basin, Eastern Silurian-Devonian, and Northern High Plains aquifers having the highest number of well records within their boundaries, their larger size allows for more dispersion. Consequently, these aquifers exhibit a lower density of well records per square kilometer.

### Supplementary information

Table S1

Table S2

Table S3

Table S4

Table S5

Table S6

Table S7

Table S8

Table S9

Table S10

## Data Availability

All codes for data standardization are available at the HydroShare data respository^11^.
